# Clinical Characteristics and Risk Factors for Severe Outcomes of Novel Coronavirus Infection, January–March 2020, Japan

**DOI:** 10.2188/jea.JE20200519

**Published:** 2021-08-05

**Authors:** Yuuki Tsuchihashi, Yuzo Arima, Takuri Takahashi, Kazuhiko Kanou, Yusuke Kobayashi, Tomimasa Sunagawa, Motoi Suzuki

**Affiliations:** Infectious Disease Surveillance Center, National Institute of Infectious Diseases (NIID), Tokyo, Japan

**Keywords:** surveillance, SARS-CoV-2, clinical characteristic, risk factor, epidemiology

## Abstract

**Background:**

Notifications of novel coronavirus infections increased in early 2020 in Japan. We described characteristics of novel coronavirus infection cases and analyzed risk factors for severe outcomes.

**Methods:**

Cases were persons with laboratory-confirmed novel coronavirus infection reported under national surveillance between January and March 2020. Clinical characteristics were described, and risk factors of (1) intensive care unit [ICU] admission and (2) invasive ventilation/death were analyzed using Poisson regression.

**Results:**

Among the 516 cases analyzed, median age was 60 years (range: 1–97 years) and 285 (55%) were male. Common symptoms/signs were fever (375/475, 79%), cough (353/465, 76%), and pneumonia (245/387, 63%). Ten (2%) cases died. Of the 348 cases with data, 50 (14%) required invasive ventilation. Adjusted for each other, male gender and 1-year increase in age were associated with ICU admission (risk ratio [RR] 4.18; 95% confidence interval [CI], 1.69–10.32 and RR 1.05; 95% CI, 1.03–1.08, respectively) and invasive ventilation/death (RR 2.79; 95% CI, 1.49–5.21 and RR 1.06; 95% CI, 1.04–1.08, respectively). Diabetes, dyslipidemia, hyperuricemia, and lung diseases were also associated with severe outcomes. Of the 80 cases asymptomatic at hospitalization, 40 developed symptoms and five of them >70 years of age required invasive ventilation.

**Conclusions:**

The early stage of the novel coronavirus epidemic in Japan disproportionately affected the elderly. Older age, male gender, and underlying conditions were associated with severe outcomes. Notably, some elderly case-patients who were asymptomatic at diagnosis and promptly hospitalized still went on to develop severe disease, indicating the importance of careful monitoring of certain populations.

## INTRODUCTION

Following the report of a novel coronavirus infection (severe acute respiratory syndrome coronavirus 2 [SARS-CoV-2]) in Wuhan City, China, in December 2019,^1^^,^^2^ a patient with pneumonia who had been in Wuhan was reported as the first confirmed case in Japan on January 15, 2020.^3^ Subsequently, additional cases were reported in Japan in January,^4^ and from late January to February, cases were detected among returnees from Wuhan on chartered flights^5^^,^^6^ and crew members and passengers on the Diamond Princess cruise ship (3,711 persons aboard).^7^^–^^10^ All persons from the chartered flights and the cruise ship underwent polymerase chain reaction (PCR) testing, regardless of sign/symptom presence.^5^^,^^6^^,^^8^^,^^10^

From early March, the number of cases suspected of being infected overseas increased, and from mid-March, sporadic cases with an unknown source of infection began to increase. In late March, multiple clusters of cases were reported, mainly from urban areas, further increasing the number of reported cases. In response, the Japanese government issued an emergency declaration to seven urban prefectures on April 4, and all 47 prefectures on April 16.^11^ With the rapid rise in the number of reported cases, including severe cases and many cases that could not be linked epidemiologically, there was concern of the healthcare system becoming overwhelmed. Therefore, in late April, local governments started to implement a variety of measures tailored to their local situation and context.

In the present study, to shed light on the epidemiology of coronavirus infection in the early phase of the epidemic in Japan (January–March, 2020), we described the clinical characteristics of novel coronavirus infection and evaluated the frequency of and risk factors for severe outcomes (intensive care unit [ICU] admission or invasive ventilation/death). Importantly, universal PCR testing of all returnees from Wuhan on chartered flights^6^ and persons onboard the cruise ship Diamond Princess^10^ enabled us to follow-up cases that were asymptomatic at the time of detection.

## METHODS

### Data collection

Novel coronavirus disease (COVID-19) was added to the list of designated infectious diseases under the Act on the Prevention of Infectious Diseases and Medical Care for Patients with Infectious Diseases (the Infectious Diseases Control Law) on February 1, 2020. This law requires a physician who diagnoses a novel coronavirus infection to immediately notify the local public health center (PHC) of the diagnosis. The PHC entered the collected data into the National Epidemiological Surveillance of Infectious Diseases (NESID) system.^12^ During the study period, all persons diagnosed with laboratory-confirmed (PCR test and/or virus isolation) novel coronavirus infection, regardless of sign/symptom presence, were hospitalized, with two consecutive negative PCR tests required for discharge.

From the NESID system, the following data were collected: name, NESID ID, age, gender, onset date, signs/symptoms at diagnosis, testing method, test results, suspected infection route, and the local jurisdiction that reported the case.

Additionally, PHCs worked with the hospitals to follow-up the cases after they were hospitalized and reported the clinical status daily to the Novel Coronavirus Response Headquarters team at the Ministry of Health, Labour and Welfare (MHLW), under the Infectious Diseases Control Law. The data collected were the following: name, NESID ID, age, gender, underlying diseases, onset date, daily symptoms, hospital admission date, discharge date, type of care provided (ICU admission, ventilation), and clinical outcome.

**Figure 1.**
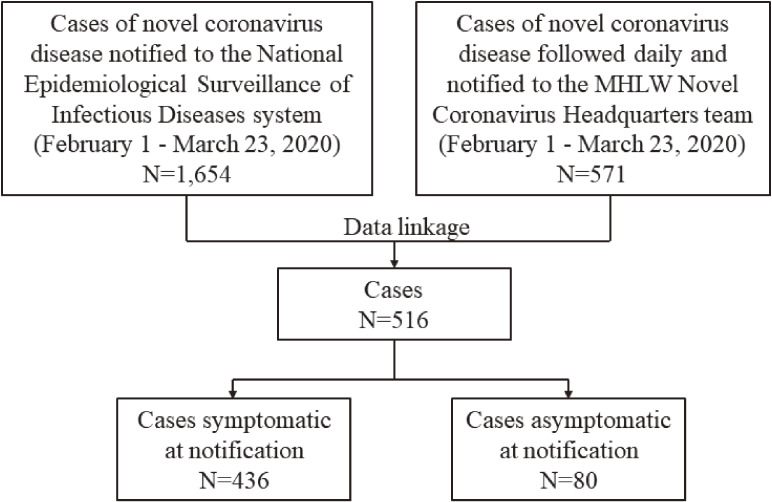
Flow diagram. MHLW, Ministry of Health, Labour and Welfare

The cases reported to the NESID system at diagnosis were linked to the hospitalized cases by PHCs using the following data: name, NESID ID, age, gender, and/or jurisdiction; We analyzed the confirmed, linked cases reported between February 1 and March 23, 2020 (Figure 1).

### Statistical analysis

We first described the demographic and clinical features of the cases. Univariate and multivariable analyses were performed to evaluate the relationships between the clinical characteristics and severe outcomes (ICU admission or invasive ventilation/death). Poisson regression with robust standard errors were used to estimate risk ratios (RRs) and their 95% confidence intervals (CIs).^13^ In using Poisson regression analysis, we assessed the mean and variance of the outcome (ie, ICU admission or invasive ventilation/death) and the over/under-dispersion of the Poisson distribution assumption using the Kolmogorov-Smirnov test.^14^ For the multivariable analyses, age was treated as a continuous variable, and gender and underlying diseases were treated as dichotomous variables. Additionally, the relationship between the number of underlying diseases and severe outcomes were evaluated by univariate and age-adjusted analyses, stratified by gender; the Wald test was used to assess for a linear dose-response trend. Last, cases who were asymptomatic at diagnosis were followed up for outcomes.

Categorical variables were described as counts and percentages. Continuous variables were described using mean, median, interquartile range (IQR), and range values. *P* < 0.05 (two-sided) was considered statistically significant. SPSS 23.0 for Windows (IBM, Inc., Armonk, NY, USA) was used to analyze the data.

### Ethics

This study was conducted under Articles 12 and 15 of the Infectious Diseases Control Law in Japan for public health purposes and was thus exempt from ethical review.

## RESULTS

### Demographics

A total of 1,654 cases were notified to the NESID system during the study period. Five hundred and seventy-one cases were monitored daily following their diagnosis through March 23, 2020; 516 of these cases were linked to cases reported to the NESID system (Figure 1), including 214 cruise ship-related cases (crew and passengers), 9 returnees from Wuhan on chartered flights, and 293 others. All 516 cases were legally hospitalized, and of the 476 cases who had symptoms at diagnosis (*n* = 436) or developed symptoms after hospitalization (*n* = 40) (Figure 1), the date of onset was known for 405 (occurring between January 20 and March 21).

Of these 516 cases, 285 (55%) were men. Their median age was 60 years (range, 1–97 years) and 51% (263/516) were aged ≥60 years (Table 1). Among the cruise ship-related and chartered flight-related cases, 115 (54%) and 7 (78%) were men, and their median age was 70 years (range, 20–91 years) and 50 years (range, 41–77 years), respectively. Excluding four cases with unconfirmed nationality, the nationalities of the cases were as follows: 405 Japanese, 25 American, 20 Australian, 13 Chinese, 9 Canadian, 9 Filipino, 7 Indian, and 12 other nationalities each with ≤5 cases.

**Table 1.** Characteristics (gender, age, and underlying diseases) of study subjects who required ICU admission or invasive ventilation, or who died

		All		ICU Admission		Invasive ventilation		Death	

		*n*	%	*n*	%	*n*	%	*n*	%
Gender	Male	285/516	55	30/182	16	42/198	21	6/285	2
	Female	231/516	45	5/141	4	8/150	5	4/231	2
Age, years	≥60	263/516	51	31/154	20	46/166	28	10/263	4
	0–59	253/516	49	4/169	2	4/182	2	0/253	0
Diabetes		39/332	12	6/31	19	13/34	38	4/39	10
Hypertension		61/332	18	7/44	16	12/49	24	2/61	3
Dyslipidemia		31/332	9	5/23	22	10/26	38	0/31	0
Hyperuricemia		8/332	2	2/5	40	2/5	40	0/8	0
Asthma		12/332	4	1/9	11	1/10	10	0/12	0
Bronchitis		3/332	1	0/2	0	0/2	0	0/3	0
COPD		3/332	1	0/2	0	1/2	50	0/3	0
Emphysema		1/332	0.3	—	—	1/1	100	0/1	0
Tuberculosis		1/332	0.3	—	—	—	—	0/1	0
Cancer (including lung cancer)		22/332	7	3/16	19	5/18	28	3/22	14
Lung cancer		5/332	2	1/2	50	2/4	50	1/5	20
Lung diseases (Asthma/Bronchitis/COPD/ Emphysema/Tuberculosis/Lung cancer)		25/332	8	2/15	13	5/19	26	1/25	4
Lung diseases (COPD/Emphysema/ Tuberculosis/Lung cancer)		10/332	3	1/4	25	3/7	43	1/10	10
Cardiovascular disease		22/332	7	3/12	25	5/14	36	1/22	5
Cerebrovascular disease		6/332	2	1/4	25	1/5	20	1/6	17
Vascular diseases (cardiovascular and cerebrovascular)		25/332	8	3/14	21	5/16	31	2/25	8
Thyroid disorder		8/332	2	0/7	0	0/7	0	1/8	13
Liver disease		6/332	2	0/5	0	0/5	0	0/6	0
Renal disease		6/332	2	0/5	0	0/5	0	0/6	0
Gastrointestinal disease		9/332	3	1/7	14	1/8	13	0/9	0
Autoimmune disease		3/332	1	0/2	0	0/2	0	0/3	0
Dementia		6/332	2	0/5	0	0/5	0	0/6	0

COPD, chronic obstructive pulmonary disease; ICU, intensive care unit.

The denominator values vary due to missing or unknown information regarding the symptom or medical intervention.

The median time from the reported date of infection to the date of onset of symptoms (ie, incubation period) was 4.0 (IQR, 2.0–6.0) days (*n* = 109), that from the onset date to the diagnosis date was 6.0 (IQR, 3.0–9.0) days (*n* = 390), and that from the diagnosis date to the date of death among those who died during the follow-up period was 12.0 (IQR, 9.5–16.5) days (*n* = 9). For the discharged patients, the median duration of hospitalization was 16.0 (IQR, 10.0–22.0) days (*n* = 226, excluding cases who died).

### Symptoms

From the time a case was reported until March 23, the main symptoms identified were fever (375/475 [79%]), cough (353/465 [76%]), general malaise (182/389 [47%]), sore throat (115/393 [29%]), nasal discharge and/or congestion (79/321 [25%]), headache (71/301 [24%]), diarrhea (65/336 [19%]), joint and/or muscular pain (42/310 [14%]), nausea and/or vomiting (20/318 [6%]), and conjunctival congestion (6/283 [2%]).

### Severe outcomes and medical intervention

Pneumonia was common (245/387 [63%]), followed by acute respiratory distress syndrome (ARDS) (10/277 [4%]), and death (10/516 [2%]). Regarding treatment, >10% of the cases required invasive ventilation (such as endotracheal intubation) (50/348 [14%]) or ICU admission (35/323 [11%]) (Table 1). Extracorporeal membrane oxygenation (ECMO) was used in 18 cases, of which two were aged 40–49 years, five were aged 60–69 years, nine were aged 70–79 years, and two were aged 80–89 years. Fifty-five patients experienced ARDS, ICU admission, invasive ventilation, ECMO, or death. Among the cases with pneumonia, the proportion of severe outcomes (requiring invasive ventilation or death) varied by age, with the highest risk for those aged ≥60 years: 0/26 (0.0%) among those aged <40 years, 2/22 (9.1%) among those aged 40–49 years, 1/37 (2.7%) among those aged 50–59 years, 15/47 (31.9%) among those aged 60–69 years, 25/52 (48.0%) of those aged 70–79 years, and 3/13 (23.0%) among those aged ≥80 years.

**Figure 2.**
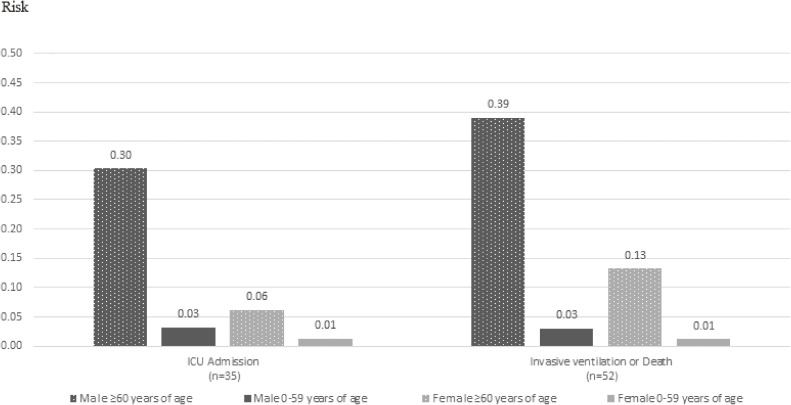
Risks of ICU admission, invasive ventilation or death among reported cases, by gender and age

Regardless of gender, being ≥60 years of age increased the risks of ICU admission and invasive ventilation/death; and, regardless of age, men had a greater risk of ICU admission and invasive ventilation/death (Figure 2). Men aged ≥60 years had the highest absolute risks, for both ICU admission and invasive ventilation/death.

### Risk factors for severe outcomes

Half of the reported cases had underlying diseases (169/332 [51%]). The three most common underlying diseases were diabetes, hypertension, and dyslipidemia (Table 1), with 91 having at least one of these three diseases. Approximately a third of the cases with these conditions required invasive ventilation. Although few, all of those with chronic obstructive pulmonary disease (COPD), emphysema, or lung cancer developed pneumonia, and a high proportion required invasive ventilation. Many of the cases with vascular disease (cardiovascular or cerebrovascular) also developed pneumonia and required invasive ventilation.

In univariate analysis (Table 2), male gender, older age, diabetes, dyslipidemia, and hyperuricemia were associated with ICU admission. These variables, along with hypertension, cancer (including lung cancer), lung diseases (COPD, emphysema, tuberculosis [TB], or lung cancer), and vascular diseases (cardiovascular or cerebrovascular), were also associated with invasive ventilation/death. Regarding vascular diseases, this association remained similarly strong when restricted to cardiovascular diseases (RR 3.40; 95% CI, 1.68–6.88).

**Table 2.** Crude and adjusted risk ratios for severe outcomes for each potential risk factor

Risk factor	ICU admission		Invasive ventilation or death	

	Crude RR (95% CI)	Adjusted Model^a^ aRR (95% CI)	Crude RR (95% CI)	Adjusted Model^a^ aRR (95% CI)
Gender, male	**4.65 (1.85–11.67)**	**4.18 (1.69–10.32)**	**3.22 (1.67–6.22)**	**2.79 (1.49–5.21)**
Age, 1-year increase	**1.06 (1.03–1.08)**	**1.05 (1.03–1.08)**	**1.06 (1.04–1.09)**	**1.06 (1.04–1.08)**
Diabetes	**2.65 (1.12–6.26)**	1.53 (0.67–3.52)	**4.27 (2.42–7.54)**	**2.48 (1.44–4.28)**
Hypertension	2.18 (0.95–5.04)	0.95 (0.37–2.39)	**2.49 (1.36–4.56)**	1.00 (0.50–2.00)
Dyslipidemia	**2.90 (1.18–7.14)**	1.85 (0.77–4.44)	**3.61 (1.97–6.62)**	**2.12 (1.15–3.91)**
Hyperuricemia	**4.90 (1.55–15.52)**	**4.01 (1.19–13.54)**	**3.12 (1.02–9.54)**	3.19 (0.95–10.69)
Cancer (including lung cancer)	2.31 (0.76–6.99)	1.11 (0.39–3.10)	**2.26 (1.00–5.10)**	1.01 (0.46–2.21)
Lung diseases (Asthma/Bronchitis/COPD/ Emphysema/Tuberculosis/Lung cancer)	1.57 (0.40–6.08)	1.14 (0.34–3.77)	2.13 (0.94–4.84)	1.35 (0.59–3.10)
Lung diseases (COPD/Emphysema/ Tuberculosis/Lung cancer)	2.93 (0.51–16.78)	1.86 (0.45–7.66)	**4.70 (2.29–9.64)**	**2.65 (1.24–5.64)**
Vascular diseases (cardiovascular and cerebrovascular)	2.66 (0.89–7.93)	1.08 (0.32–3.68)	**3.38 (1.72–6.62)**	1.33 (0.62–2.85)

aRR, adjusted risk ratio; CI, confidence interval; COPD, chronic obstructive pulmonary disease; ICU, intensive care unit; RR, risk ratio.

^a^Adjusted model: for age and gender, each is adjusted for the other; for the underlying diseases, these are adjusted for age and gender.

Adjusted for each other, gender and age were strong risk factors for a severe outcome (Table 2). In addition, when the number of underlying diseases was added to this model, gender and age remained as strongly associated with severe outcomes. Specifically, the RR for ICU admission was 3.50 (95% CI, 1.25–9.78) for male gender and 1.05 (95% CI, 1.02–1.08) for age, and the RR for invasive ventilation/death was 2.28 (95% CI, 1.16–4.46) for male gender and 1.06 (95% CI, 1.03–1.08) for age. Regarding underlying diseases, when adjusted for gender and age, diabetes, dyslipidemia, and lung diseases (COPD, emphysema, TB, or lung cancer) were associated with a greater than two-fold increase in the risk of invasive ventilation/death; hyperuricemia also increased the risk of ICU admission (Table 2). When age was treated dichotomously (≥60, 0–59 years), the results remained similar with male gender and underlying diseases all being strong risk factors for severe outcomes.

Regardless of the type of underlying disease, in univariate analysis, having one or more of these conditions was associated with a higher risk of a severe outcome; the association between having an underlying disease and a severe outcome was modified by gender, with a particularly strong association in men (Table 3). Adjusted for age, “having two or more underlying diseases” was associated with invasive ventilation/death in men; the adjusted RR was 3.99 (95% CI, 1.02–15.55). In the age-adjusted model, the test for linear trend for the number of underlying diseases was not significant in men (*P* = 0.43 for ICU admission, *P* = 0.14 for invasive ventilation/death) or in women (*P* = 0.62 for ICU admission, *P* = 0.58 for invasive ventilation/death).

**Table 3.** Crude and adjusted risk ratios for severe outcomes, by the number of underlying diseases, stratified by gender

Number of underlying diseases	ICU admission		Invasive ventilation or death	

	Crude RR (95% CI)	Adjusted Model^a^ aRR (95% CI)	Crude RR (95% CI)	Adjusted Model^a^ aRR (95% CI)
Men				
≥2	**5.53 (1.59–19.20)**	2.52 (0.59–10.79)	**8.97 (2.77–29.05)**	**3.99 (1.02–15.55)**
1	**5.38 (1.45–19.97)**	2.48 (0.57–10.76)	**7.31 (2.09–25.55)**	3.40 (0.83–13.90)
0	1	1	1	1

Women				
≥2	1.22 (0.12–12.86)	0.35 (0.04–2.99)	4.64 (0.90–23.90)	0.74 (0.17–3.29)
1	1.13 (0.11–11.93)	0.73 (0.09–5.92)	3.36 (0.59–19.06)	1.63 (0.37–7.25)
0	1	1	1	1

aRR, adjusted risk ratio; CI, confidence interval; ICU, intensive care unit; RR, risk ratio.

^a^Adjusted model: Adjusted for age in years (continuous variable).

### Asymptomatic cases

The number of asymptomatic cases at the time of initial diagnosis was 80/516 (16%). By March 23, 40/80 cases (50%) had developed symptoms, including pneumonia in 20 patients. Of these 40 patients, 11 had the onset date recorded, and the median time period between diagnosis and onset was 1.0 (IQR, 1.0–6.0) day. The mean age of the 40 cases that developed symptoms after diagnosis (ie, symptomatic cases) and the 40 cases that remained asymptomatic were 64.3 and 58.3 years, respectively. Among the symptomatic cases, 18 (45%) were male, and of those who remained asymptomatic, 15 (38%) were male. Last, 5/40 symptomatic cases (13%), all of whom were >70 years of age, required invasive ventilation. Even in those initially diagnosed as asymptomatic, the proportion of cases requiring invasive ventilation after onset was similar to the overall proportion of cases who required invasive ventilation (50/348 [14%]).

## DISCUSSION

In this study, we investigated the demographic and clinical characteristics of 516 case-patients with novel coronavirus infection identified by national surveillance, along with the risk factors associated with severe outcomes, during the early stage of the epidemic in Japan. Of the 516 case-patients, approximately half were men aged ≥60 years and ∼80% had fever or cough. Nearly two-thirds of the cases had pneumonia, >10% required invasive ventilation, and 10 died. Male gender, older age, and underlying diseases, particularly lifestyle-related diseases (eg, diabetes, dyslipidemia, and hyperuricemia), and lung diseases, were associated with severe outcomes. In men, in addition to the strong association with severe outcomes, the effect of having an underlying disease was much larger than in women. Notably, half of the cases who were asymptomatic at the time of hospitalization developed disease during follow-up, and a proportion of them developed severe outcomes. The potential seriousness of COVID-19 was confirmed because rapid deterioration occurred during medical monitoring; such outcomes could not be attributed to delays in obtaining healthcare. This finding illustrates the challenges in preventing severe COVID-19 in some patients, even when the disease is diagnosed early with prompt hospitalization.

These results are in general consistent with other reports. The median incubation period was 4 days, similar to that reported in China.^15^ While the majority of case-patients in this study were those suspected of having COVID-19 and tested, and the full clinical spectrum of COVID-19 could not be determined, fever, cough, and general malaise were common, as reported by others.^16^^–^^20^ With regard to the proportion of severe outcomes, 5.0% required ICU admission, 2.3% required invasive ventilation, and 1.4% died in a study of 1,099 cases (median age 47 years) in China.^15^ Our study in the early phase of the epidemic in Japan had higher proportions of severe outcomes among the medically-attended cases than that in China’s study. However, this may be due to the fact that the population was older (median age 60 years) and/or differences in the testing intensity or medical protocols. Regarding underlying diseases, the results were similar with those of previous studies. In a study of 44,672 cases in China, the case fatality rate was reported to be higher in patients with underlying diseases (cardiovascular disease, diabetes, chronic respiratory disease, hypertension, or cancer).^21^ Similarly, various other studies have suggested that older age, men, obesity, smoking, hypertension, diabetes, cardiovascular disease, chronic lung disease, and chronic kidney disease are risk factors for severe outcomes.^18^^,^^19^^,^^22^^–^^26^

Although exploratory and limited in sample size, the data suggested that dyslipidemia and hyperuricemia may be risk factors for severe outcomes, in addition to some previously documented factors of age, male gender, diabetes, and lung diseases. Although some have reported dyslipidemia as a risk factor for serious outcomes,^27^ few studies have suggested an association with hyperuricemia.^28^ Pathologically, SARS-CoV-2 infection has been suggested to affect not only parenchymal epithelial cells in the lung but also endothelial cells throughout the body, inducing endotheliitis.^29^^,^^30^ Male gender, hypertension, diabetes, and all other conditions associated with pre-existing endothelial dysfunction, are important factors for severe outcomes of novel coronavirus infection.^29^^,^^30^ Dyslipidemia is a risk factor for pre-existing endothelial dysfunction,^31^ and hyperuricemia may induce endothelial dysfunction through increase in inflammation and oxidative stress.^32^ Additionally, the present findings suggest that lung diseases (COPD, emphysema, TB, and lung cancer) are also risk factors for severe outcomes, potentially due to possible pre-existing destruction of lung structures.

Notably, half of the case-patients who were asymptomatic at the time of diagnosis developed symptoms/signs (including 20 case-patients who developed pneumonia) after admission, and 5/40 (13%) required invasive ventilation. Although many asymptomatic cases have been reported,^33^ studies that have followed up such cases have been limited and small in sample size.^34^^–^^39^ In China, 17/24 cases (70%) asymptomatic at detection developed symptoms or abnormal findings on computed tomography scans, although none of them developed severe outcomes.^34^ In Italy, it was reported that mild symptoms developed in 2/5 (40%) asymptomatic cases,^35^ whereas in New York, 10/14 (71%) asymptomatic pregnant women developed disease, of which two (14%) required admission to the ICU.^36^ While such differences can arise from many reasons (eg, the intensity of testing, age group tested), it was notable in this study that 40 of those who were diagnosed when asymptomatic went on to develop symptoms, with five cases aged >70 years requiring invasive ventilation. These cases that were asymptomatic at diagnosis comprised 10% of the reported cases,^40^ and were tested because they were close contacts of confirmed cases or due to their occupation or the contextual situation (eg, disembarkation from a cruise ship). Given these results, even if asymptomatic at the time of diagnosis, it is important to closely monitor certain individuals, such as elderly men and those with underlying lifestyle diseases or lung disease.

This study had several limitations. First, only 516 cases could be followed up, although 1,654 cases were notified through NESID; cases that were followed versus those that were not may have introduced selection bias. However, the distribution of gender, age, and symptoms at the time of diagnosis were similar between these two groups (eTable 1). The NESID system is currently not used for COVID-19 surveillance because the local governments have incrementally switched to a new, dedicated COVID-19 surveillance system, “HER-SYS (Health Center Real-time Information-sharing System” on COVID-19), from May 22, 2020. Follow-up of case-patients by the MHLW’s Novel Coronavirus Response Headquarters team has also been switched to HER-SYS. HER-SYS, however, is still being refined as of March 2021. Second, because non-response and unknown values for symptoms were excluded from the denominator of cases, the proportions calculated for each symptom may be an overestimation (while there was yes/no reporting for a symptom, a blank record was treated as missing and not counted in the denominator); the same limitation applies to the outcome data, and the risk factor results may be biased; however, our key findings were broadly consistent with reports from elsewhere. Additionally, at that time, those who had suspected symptoms of COVID-19, such as fever or respiratory symptoms, were more likely to be examined by a PCR test. It is thus important to note that the clinical spectrum of our study subjects do not represent the symptom or severe outcome distribution of SARS-CoV-2-infected persons, but those of diagnosed and reported cases. Third, given the relatively small number of cases for some of the underlying risk factors, we only adjusted for age and gender to evaluate their associations with key outcomes. However, a strength of the study was that PCR tests were performed on many asymptomatic persons, which allowed us to follow-up many asymptomatic cases who were immediately hospitalized upon viral detection; these cases confirmed the potential seriousness of COVID-19’s natural history because we could remove the effect of late healthcare-seeking behaviors. As cases were not necessarily systematically followed, censoring may have under-ascertained the occurrence of symptoms and severe outcomes also for these cases that were initially asymptomatic at diagnosis. Lastly, local incidence of COVID-19 could have been a confounder for the exposures of interest and severe outcome. For instance, if there were an increase in incidence in a certain area, it is conceivable that healthcare access/care would be compromised, leading to severe outcomes such as invasive ventilation or death among the elderly. If there was an association between age and high incidence area, this could lead to confounding. We believed this to be highly unlikely, given that the background infection situation was still relatively low in Japan. In fact, when we accounted for PHC or prefecture as a nuisance parameter and estimated odds ratios using conditional logistic regression, the results remained qualitatively the same and confirmed that the effects of prefecture or PHC were limited during this period.

In conclusion, old age, male gender, and underlying diseases were associated with more severe COVID-19 disease. In particular, men aged ≥60 years and patients with lifestyle-related diseases or lung diseases were at higher risk of requiring critical medical interventions. As some case-patients who were hospitalized while asymptomatic went on to develop severe outcomes, we iterate the importance of careful monitoring for certain risk groups.

## APPENDIX A. SUPPLEMENTARY DATA

eTable 1. Comparison between subjects in this study vs. cases notified through the NESID system that could not be linked to follow-up data (i.e., cases not included in this study)
