# Supplementary material for: Clinical Characteristics and Risk Factors for Severe Outcomes of Novel Coronavirus Infection, January–March 2020, Japan
Source: J Epidemiol. 2021 Aug 5;31(8):487–94. doi: 10.2188/jea.JE20200519 (PMC8275442; doi:10.2188/jea.JE20200519)
Supplement: Supplementary file 1 [file je-31-487-s001.pdf]

eTable 1. Comparison between subjects in this study vs. cases notified through the NESID system that could not be linked to follow-up data (i.e., cases not included in this study)

		Subjects in this study (n=516) n (%)	Cases notified through NESID that could not be linked to follow-up data (n=1,138) n (%)	p-value
Gender	Male	285 (55.2%)	615 (54.0%)	0.69
	Female	231 (44.8%)	523 (46.0%)	
Age, years	≥60	263 (51.0%)	579 (50.9%)	0.98
	0–59	253 (49.0%)	559 (49.1%)	
Sign/symptom	fever	323 (62.6%)	706 (62.0%)	0.87
at time of diagnosis	cough	226 (43.8%)	420 (36.9%)	0.01
	severe pneumonia	33 (6.4%)	90 (7.9%)	0.32

NESID, National Epidemiological Surveillance of Infectious Diseases.
